# Electrowetting on glassy carbon substrates†

**DOI:** 10.1039/d4na00506f

**Published:** 2024-08-30

**Authors:** Sittipong Kaewmorakot, Athanasios A. Papaderakis, Robert A. W. Dryfe

**Affiliations:** a Henry Royce Institute, University of Manchester Oxford Road Manchester M13 9PL UK; b Department of Chemistry, University of Manchester Oxford Road Manchester M13 9PL UK robert.dryfe@manchester.ac.uk

## Abstract

The wetting properties of carbon surfaces are important for a number of applications, including in electrochemistry. An under-studied area is the electrowetting properties of carbon materials, namely the sensitivity of wetting to an applied potential. In this work we explore the electrowetting behaviour of glassy carbon substrates and compare and contrast the observed response with our previous work using highly oriented pyrolytic graphite. As with the graphite substrate, “water-in-salt” electrolytes are found to suppress faradaic processes, thereby enlarging the electrochemical potential window. A notable difference in response to positive and negative polarity was seen for the graphite and glassy carbon substrates. Moreover, whereas graphite has previously been shown to give a reversible electrowetting response over many cycles, an irreversible wetting was observed for glassy carbon. Similarly, the timescales of the wetting process were much faster on the graphitic substrate. Reasons underlying these marked changes in behaviour on the different carbon surfaces are suggested.

## Abstract 2

Electrowetting on a glassy carbon substrate is demonstrated. The process is irreversible, in contrast to earlier observations of reversible wetting seen with basal plane graphite.

## Graphical abstract

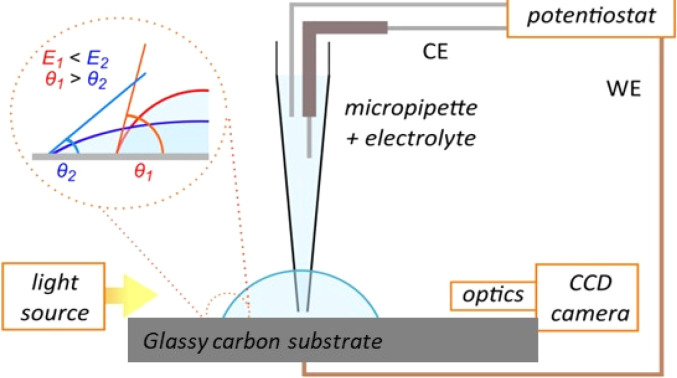

## Introduction

The contact angle of a liquid droplet on a solid surface is a macroscopic measure of the microscopic interactions at the solid/liquid interface.^1^ Surface free energy is minimized by the droplet spreading on surface. Then, the drop maintains its equilibrium state with a spherical cap and contact angle (CA) described by the well-known Young–Dupré equation.^2^1
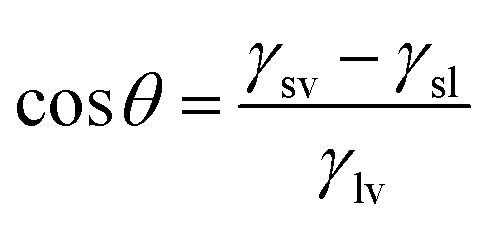
where *γ*_sv_, *γ*_sl_, and *γ*_lv_ refer to solid-vapor, solid–liquid, and liquid–vapor interface tension (or surface tension) respectively.

Charging of the solid *via* application of a potential offers a route to vary the solid–liquid term above. This “electrowetting” phenomenon was first indirectly reported *via* the electrocapillary effect at the mercury/electrolyte interface by Gabriel Lippmann^3,4^ in 1875; Lippmann interpreted the variation of the position of the meniscus at the mercury/electrolyte interface with applied potential in terms of the change in the interfacial energy. Electrowetting did not gain much immediate attention, due to the inability to extend the wetting effect to solid conductors, largely due to the difficulties in preparing atomically smooth surfaces (unlike the case of mercury) and the associated pinning of the expanding droplet on such defects. There are further side effects, such as the decomposition of electrolytes and corrosion of substrates, which pose additional challenges. Consequently, this interesting phenomenon was rather overlooked. Half a century after Lippmann's work, Frumkin *et al.* performed experiments on oil droplets on mercury electrodes immersed in different electrolytes to study the electrochemical double layer formed spontaneously after the applied potentials deviated from potential of zero charge (*E*_pzc_).^5^ Accumulation of charge at the interface led to the decrease of interfacial tension, macroscopically detected as changes in the contact angle of the oil droplet. This study was a pioneering one, which built on Lippmann's theory of electrocapillary.^6^2
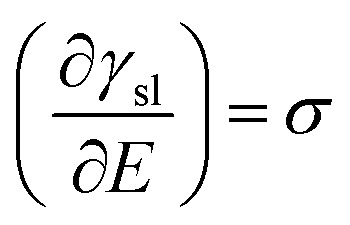
where *σ* represent charge per unit area of the polarizable interface, *E* refers to potential, under the assumption that temperature, pressure and chemical potential are constant. The potential dependence of interfacial tension could be determined by integrating eqn (2) from the potential of zero charge (pzc) to any applied potential bias, followed by substitution of the solid–liquid interfacial tension after integration of the Lippmann eqn (2) into the Young–Dupré at the three-phase contact line (TCL). Hence, the resultant Young–Lippmann equation (Y–L) could be used to predict the CA dependence on applied potential, when the latter deviates from the potential of zero charge, yielding a quadratic relationship as shown in following equation where *C* denotes the capacitance of the interface.3
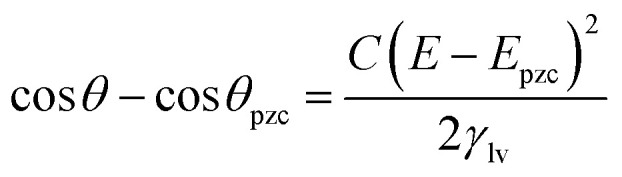


Interest in electrowetting returned with the introduction of a dielectric layer, usually of polymer, to insulate the solution phase from the solid substrate, the so-called electrowetting on dielectric (EWOD) configuration, so as to control the droplet movement in lab-on-a-chip devices. Although tens to hundreds of Volts was required as an energy input to accomplish CA changes of 70^0^,^7–9^ the advantage of this approach is that the side reactions occurring at high potentials are suppressed. However, by integration of the insulating layer capacitance term in the Y–L equation, *C*, would be approximately equal to *ε*_0_·*ε*_d_/*d* where *ε*_d_ is the dielectric constant of the dielectric layer (relative permittivity), and *d* is the thickness of the insulating polymeric overlayer. The EWOD system actually contains two capacitors in series, but the capacitance of the solid–insulator interface can be neglected because the thickness of the polymer layer is normally much larger than the closest approach of the ions to the interface.^3^ Thus, the capacitance term in the EWOD system can be treated as being independent of potential, even though the system was studied on an ideally polarizable electrochemical interface.^10^

Very recently, our laboratory has described an alternative approach, namely that fully reversible electrowetting can be achieved with electrolytes placed directly on the surface of conductors: this is known as electrowetting-on-conductor (EWOC).^10,11^ This has been achieved on the basal plane of highly oriented pyrolytic graphite (HOPG) as a substrate, and a stronger effect was noted with highly concentrated aqueous solutions (“water-in-salt” electrolytes). The definition of the term “water-in-salt” was provided by Suo *et al.* as the case where the amount of salt exceeds both weight and volume of solvent in a binary system.^12^ This type of electrolyte has attracted much attention recently in a variety of electrochemical applications especially for energy storage, owing to its cost-effectiveness, low toxicity, low flammability, and wide electrochemical window. Under this regime, the potential window of the aqueous electrolyte could be expanded to 2.6–2.8 V depending on the electrolytes^11,13,14^ because water decomposition reactions (oxygen and hydrogen evolution) can be suppressed by increasing electrolyte concentration. In addition to ion association during the increase of salt concentration, the solvent-separated ion pair interaction (SSIPs), normally present in the dilute solution, become replaced by contact ion pairs (CIPs) or aggregated cation–anion pairs (ACAPs) due to the lack of free solvent molecules.^12,15,16^ For example, as predicted by molecular dynamics calculations, the number of water molecules in the Li^+^ ion solvation sheath decreases from 11 to 6, 4, and 2.6 following an increase in the concentration of lithium bis(trifluoromethanesulfonyl) imide from 5 m to 9.3 m, 13.9 m, 20.8 m, respectively.^17^ The conductivity in the water-in-salt condition, however, decreases due to the increased viscosity of concentrated electrolytes, which plays a role in ionic conductivity, but also the ion pair interactions, *e.g.* formation of CIPs, and ACAPs.^16,18^ The electrostatic interactions and coulombic ion friction, depending on ion nature and size, are the main causes of the higher viscosity seen when increasing salt concentration.

To date, the EWOC approach has largely been illustrated on basal plane graphite, although a variety of electrolytes have been explored. Carbon materials are used in many electrochemical applications in particular, and possess interesting electronic properties.^19^ The Raman spectroscopy of these materials can be used as an indirect probe of the latter. The spectrum of graphite is dominated by a Raman shift at ≈1580 cm^−1^, representing the E_2g_ vibrational mode (stretching mode of sp^2^ carbon bond, G band) indicating the highly ordered, basal plane of graphite. Other carbon materials, including glassy carbon (GC) show another Raman band in the region of ≈1360 cm^−1^ related to structural defects or disorder within the graphite structure (the 
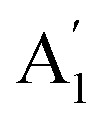
 vibrational mode or breathing of sp^2^ ring affected by sp^3^ carbon, D band),^19–21^ so the ratio between these two bands represents the sp^2^/sp^3^ hybridizations of carbon electrode materials. Consequently, graphite materials exhibit a low density of states (DOS) near the Fermi level, owing to the small overlap between valence and conduction band, while the defects associated with disordered carbons fill the DOS near the Fermi level, so the electronic behaviour of HOPG can be treated as semi-metallic, while disordered graphite and GC, behave as low DOS metals.^19,22^ In contrast, the topography of HOPG and GC surfaces (roughness) is similar, the mean roughness reported *via* AFM characterization is 1.77 nm and 3.3 nm for HOPG and GC, respectively.^23,24^ The surface termination of each material strongly affects the oxygen content: XPS data suggests that the polished GC surface contains more than 10 per cent of oxygen,^19^ while freshly exfoliated HOPG showed oxygen content below 0.5 per cent.^25^ In this work, we compare and contrast the electrowetting properties of GC with our earlier reports on graphite, again using the EWOC configuration. We maximise the electrowetting effect by exploiting the enlarged electrochemical potential window offered by the use of the water-in-salt regime, here with aqueous lithium chloride electrolyte. Furthermore, the dynamic electrowetting behaviour is also evaluated, to study the reversibility of wetting on the GC surface.

## Experimental

### Electrode preparation

GC (5.00 mm active material diameter, supplied by Pine Research Instrumentation) was used as the working electrode (WE), and polished with alumina suspension of particle size 1 μm and 0.05 μm (MicroPolish™) on TexMet C and MicroCloth polishing cloths (from Buehler) respectively, then rinsed several times with ultra-pure water. The GC substrate was attached to Cu wire (0.25 mm diameter) using Cu tape for the connection. Silver conductive epoxy (RS components, UK) was used for electrical connection between copper wire and highly oriented pyrolytic graphite HOPG, ZYA grade, mosaic spread 0.4 ± 0.1°, (from TipsNano) after 24 h of epoxy curing, the connection was then covered with insulating resin. The HOPG surface was mechanically cleaved with Scotch tape to ensure each experiment was done on a pristine surface with minimal airborne contamination. PTFE-coated silver wire (0.20 mm and 0.035 mm diameter and coating thickness, respectively, supplied by Advent, U.K.) was washed with ultra-pure water before use as a pseudo-reference electrode (RE). Pt wire (0.05 mm diameter From Advent, U.K.) was used as the counter electrode: the wire was cleaned with a butane flame immediately prior to use.

### Electrowetting setup

**Fig. 1:**
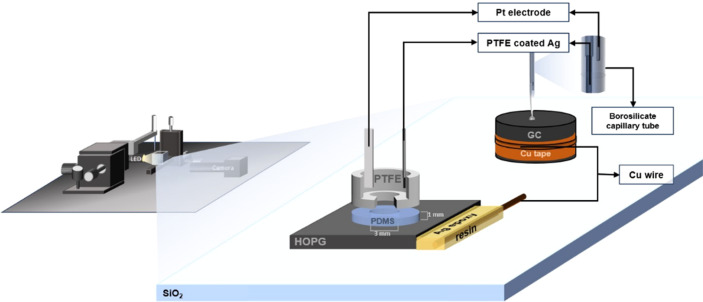
Schematic of the electrowetting and capacitance measurement set-up on GC and HOPG substrates.

The experimental set-up is shown in Fig. 1. Borosilicate glass capillary tubes (from World Precision Instruments, inner diameter 0.84 mm, outer diameter 1.5 mm and length of 100 mm) were pulled with a Flaming/Brown Micropipette Puller. The pulling parameters were previously optimized in order to obtain a micropipette with an inner dimeter of 2–3 μm. The LiCl solution was later inserted into the pulled pipette with a 28 G MicroFil Needle (also from World Precision Instruments). Both substrate and the pipette position were controlled by a 3-axis micro-positioner stage from Thorlabs (model MBT613/M). On the pipette side, a microinjector (PV820 Pneumatic PicoPump, from World Precision Instruments) was connected so as to adhere the electrolyte droplet of 0.20–0.30 mm diameter to the substrate. The substrate was placed in a special optical glass box (from Hellma Analytics) and surrounded by ultra-pure water (Millpore) for the case of dilute electrolytes so that the high humidity can compensate for evaporation of the small droplet. The images of the droplet were taken using an Infinity2 microscope camera (Microscope Optical Services) with an LED light source; the videos were recorded at a rate of 30 frames per second.

### Cell configuration for capacitance measurement

To control the exposed surface area, a Teflon-walled cell was placed on the working electrode surface (the exposed area of solution was 0.7068 cm^2^); poly(dimethyl siloxane), PDMS (Sylgard ™527, Dow Corning), was applied to the bottom of the PTFE cell to prevent solution leakage between the surface of substrate and the cell.^26^ Thus, in order to create the PDMS with a diameter to match the PTFE, the PTFE cell was flipped then mounted to the mold. Then, the PDMS mixture (with a 1 : 1 ratio of compartment A and B) was carefully added into the mold and the combination was transferred into an oven, which was held at 125 °C for 75 minutes to cure the gel. The PTFE cell, with the PDMS attached, could be removed from the mold after it was cooled to room temperature. Finally, electrolytes were injected into the cell as shown in Fig. 1.

### Electrochemical measurements

All electrowetting experiments were performed on an Ivium potentiostat (Octostat5000), operated *via* Iviumsoft software. The static electrowetting experiments were conducted by application of consecutive potential steps in the potential window of 0.0 to +1.8 *vs.* Ag wire pseudo-reference electrode on GC with a 50 mV increment potential step, while the duration of each step was 5 s. The same duration time and step potential were applied over the negative potential range, which extended from 0.0 V to −2.5 V *vs.* Ag wire. A pseudo-reference electrode was used, because we found problems with the stability of the Ag/AgCl reference electrode at high chloride concentrations. This is consistent with the known solubility of AgCl at high (molal) chloride concentrations.^27^ This, however, led to some drift in potentials between the electrowetting and impedance measurements, meaning that the comparative data (*e.g.*Fig. 5) is reported *vs.* the potential of zero charge to enable direct comparison. Note that the potential of zero charge is readily identified in both cases from the minimum in capacitance and maximum contact angle in electrowetting. Cyclic voltammetry (CV) was employed to investigate surface processes as a function of electrolyte concentration on both substrates at a scan rate of 50 mV s^−1^ in the PTFE electrochemical cell, with a 3-electrode configuration. For electrochemical impedance spectroscopy (EIS), experiments were conducted on an Autolab PGSTAT302N potentiostat in the frequency range 20 kHz to 1 Hz with a 7 mV peak-to-peak amplitude. Constant potentials were applied from 0.0 V (*vs.* Ag) to the cathodic and anodic limit of the electrolytes' potential window with a 50 mV increment.

Capacitance values were calculated from EIS data, *via* a graphical approach developed by Tribollet and colleagues,^28^ interpreting an effective capacitance (*C*_eff_) at each frequency (*f*/Hz) from the Bode plot with the following equation.4
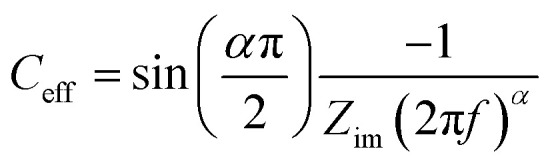
where *Z*_im_ is the imaginary part of the impedance. *α* the constant phase element exponent, which could be estimated from the slope between log(*f*) and log(*Z*_im_). The total capacitance of the interface is obtained by taking an average in the range of frequencies where the interface acts as a capacitor, *i.e.* the phase in the Bode plot is close to −90 degrees.

### CA and surface tension measurement

The CA measurement method was adopted from previous works reported by Papaderakis and colleagues:^11^ the CA was extracted from the recorded videos by using a Canny image processing algorithm for edge detection, based on the calculated image gradient from the Gaussian derivative, with MATLAB software used for extracting the droplet curvature. Once the detected points were fitted to the equation of a circle *via* a nonlinear least-squares solver function, the CA was calculated with eqn (5). In addition, the electrolytes' surface tension was measured with the pendant drop method using an optical tensiometer (Theta Lite, from Biolin Scientific) *via* the OneAttension software: the droplet shape was then fitted to the Young–Laplace equation.5
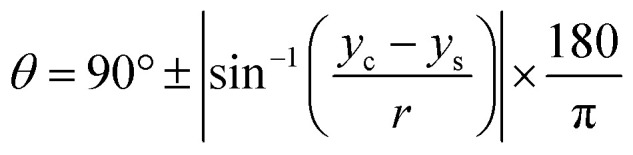
where *y*_c_ and *y*_s_ refer to coordinates on the *y* axis with respect to the circle's centre and the projection to the contact line of droplets, respectively; *r* is droplet radius.

Note that electrolyte concentrations throughout the manuscript are reported in molality units, *i.e.*, mol kg^−1^ of solvent, *m*, to account for the error introduced when using molarity (*i.e.*, mol L^−1^ of solution) in highly concentrated solutions arising from the significant contribution of the solute mass to the total mass (and hence volume) of the final solution.

## Results and discussion

**Fig. 2:**
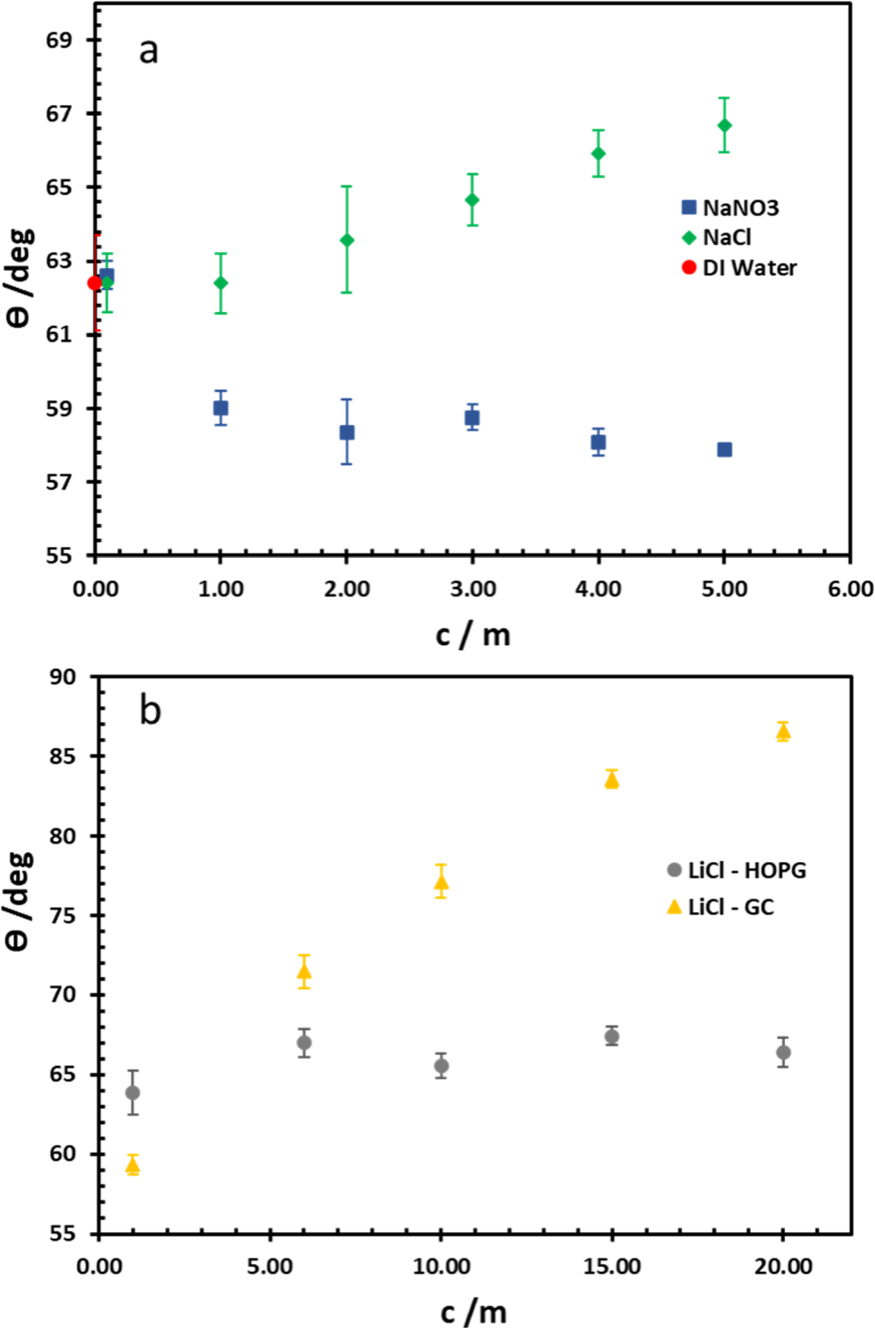
Equilibrium CA of NaCl and NaNO_3_ electrolytes on HOPG (a), and LiCl on both HOPG and GC substrates (b).

For wettability of the electrolyte on the pristine basal plane of HOPG, the graphite could be considered as a relatively hydrophilic material due to the CA of pure water (in air) being 62.4°, in agreement with earlier works (61.4°–64.0°).^14,25,29^ From Young's equation, there are two main factors that have to be taken into account as influencing the CA on a given material: surface tension (referring to the liquid/vapour interface) and the liquid–solid interfacial tension. For the higher concentrations of the electrolytes used here (NaNO_3_, NaCl, and LiCl), a higher surface tension is observed^30,31^ (for example of LiCl solutions shown in Table 1) due to the increase of electrostatic forces in solution itself, which is interpreted in terms of a negative surface excess of the ions. Consequently, in this case, the interfacial tension for NaCl and NaNO_3_ plays a crucial role on CA (Fig. 2a); the CA of NaCl increases from 62.4° to 66.7° when its concentration is increased from 0.1 m to 5 m. In contrast, a decrease in the CA of NaNO_3_ is observed from 62.6° to 57.9° while the concentration increases over the same range. Interestingly, this phenomenon agrees with the molecular dynamics simulations of Verduzco and Shen, using NaCl and NaNO_3_ droplets on a 6-layer graphene substrate:^33^ their simulations showed the density profile of ions adsorbed on the graphene; a higher density of NO_3_^−^ was predicted at the graphene interface compared to Cl^−^ at the same concentration. Hence, the interfacial tension of NaNO_3_ decreased while it increased in the NaCl case. This was interpreted in terms of NO_3_^−^ penetration, and adsorption into first dense of water layer, whereas this is harder in the case of Cl^−^ because of its comparatively stronger hydration.

**Table 1:** Surface tension and density of LiCl electrolyte as a function of concentration

LiCl concentration/m	Density (*ρ*, g cm^−3^)	Surface tension (*γ*_lv_, mN m^−1^)
20	1.30 ± 0.02	95.12 ± 0.94
15	1.23 ± 0.01	88.19 ± 0.15
10	1.18 ± 0.00	84.02 ± 0.33
5.0	1.10 ± 0.01	76.98 ± 0.94
1.0	1.01 ± 0.02	70.75 ± 1.37
Ultra-pure water	0.997^a^	70.065 ± 2.25

a Data recorded at 25 °C and quoted in ref. 32.

The wettability study of electrolyte was extended to GC (Fig. 2b), the CA of LiCl electrolyte is found to increase significantly from 59.3° to 86.5° when the salt concentration is increased from 1 m to 20 m. There is a contrast with the HOPG surface, where the CA values are unchanged, following a slight increase from 63.8° to 67.0° between the concentrations of 1 m and 5 m. On the contrary, the CA of KF droplets on HOPG, were reported to increase from 63.8° to 82.8° when the concentration increases from 0.5 M to 13 m. A couple of factors may lie behind the increase in CA of LiCl with concentration on GC, including airborne contamination of the GC surface, making it more hydrophobic. The laminar structure of HOPG means cleavage can be used to remove such contaminated layers, whereas this process is not possible with GC;^34^ also there may be an intrinsic difference in the wettability of the two materials. HOPG consists of ABAB stacking of graphene layers along the *c*-axis, which is held together by van der Waals interactions while GC has intertwined ribbons of graphitic structure,^19,35^ so there must be many voids in the structure itself. These sub-nanometre scale pores could influence the hydrophobicity of the material and also its mean roughness, which has been reported at 3.3 nm:^23^ this could be significant if the droplet wetting follows the Cassie–Baxter model^36^ given for previous reports on highly hydrophobic surfaces on the edge of HOPG, due to air pockets in the structure.^37^

**Fig. 3:**
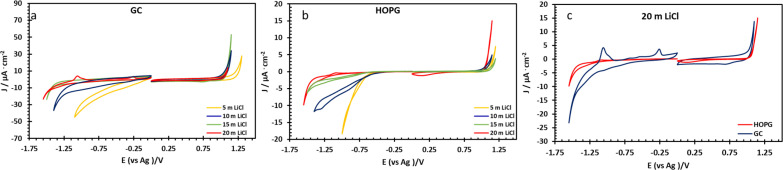
Cyclic voltammograms of LiCl solution at 5, 10, 15, and 20 m recorded on GC (a) and HOPG (b) surface in PTFE cell set up at scan rate of 50 mV s^−1^ respect to Ag pseudo-reference electrode. (c) Shows a comparison of the two electrodes at the highest concentration.

**Fig. 4:**
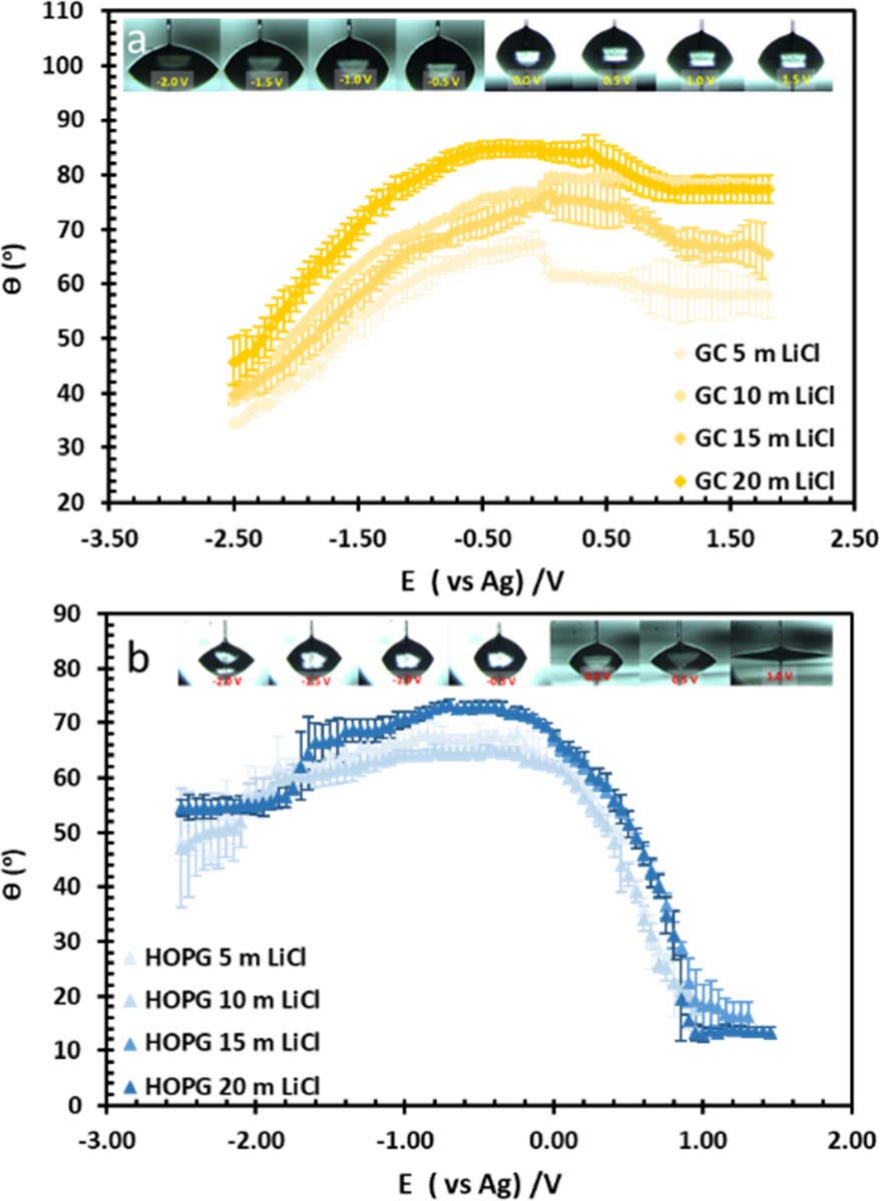
The change in CA with potential of LiCl solution at concentration of 5 m, 10 m, 15 m, and 20 m on GC (a), and HOPG (b). The droplet figures during the electrowetting experiment at 20 m LiCl shown on top of the graphs.

The electrochemical properties of highly concentrated LiCl were evaluated *via* three electrode voltammetry using GC and basal plane HOPG substrates as the working electrode. The cyclic scans were recorded from 0 V (*vs.* Ag) toward positive and negative polarization, separately, at a scan rate of 50 mV s^−1^ (Fig. 3). On both substrates, as shown in Fig. 3a and b, the magnitude of the current density (*j*) on negative polarization is seen to decrease with increasing electrolyte concentration from 5 m to 20 m; meanwhile the electrochemical potential window has expanded from approximately 1.7 V to 2.4 V (*vs.* Ag) on HOPG and from 1.4 V to 2.3 V (*vs.* Ag) on GC. This is due to the fall of the water-to-electrolyte molar ratio when solution approaches the “water-in-salt” regime: the hydrogen evolution reaction (HER) is consequently suppressed, regardless of minor pH changes.^12,15^ The voltammogram of negative polarization on GC at concentrations lower than 10 m in Fig. 3a, shows a small reduction process which can be assigned to the oxygen reduction reaction (ORR) from the presence of atmospheric oxygen. This reduction process is suppressed at higher concentrations owing to the salting-out effect, *i.e.* lower oxygen solubility in aqueous solution,^38^ and/or slower ion diffusion in more viscous of higher concentrated electrolyte.^18^ However, on the positive polarization of both working electrodes, the current density exhibits the opposite trend with LiCl concentration. Similar observations have been reported on GC with LiCl^39^ and for the HOPG surface with KF^11^ with electrooxidation of the anion (Cl^−^ in this case) occurring. Subsequent work on the HOPG surface has shown that anion intercalation can occur, as reported *via* the study of ClO_4_^−^ and TFSI^−^.^10^ Furthermore, Degoulange *et al.* also investigated two-step intercalation of halide anions on graphite surfaces: the chemical intercalation occurred after halides undergo electrochemical reduction to trihalides.^40^ We could consequently suggest that the observed electrowetting data (the constant CA after the applied potential reached 1.0 V *vs.* Ag, Fig. 4b) may be consistent with the Cl^−^ intercalation on HOPG surface proposed by Degoulange *et al.* In terms of surface topography, GC has a reported surface mean roughness of 3.3 nm (ref. 23) while it is slightly lower on HOPG (1.77 nm (ref. 24)) so the real exposure area (microscopic area) is higher on GC, which may explain the corresponding increase in current density for the comparative voltammogram in 20 m LiCl (Fig. 3c).

**Fig. 5:**
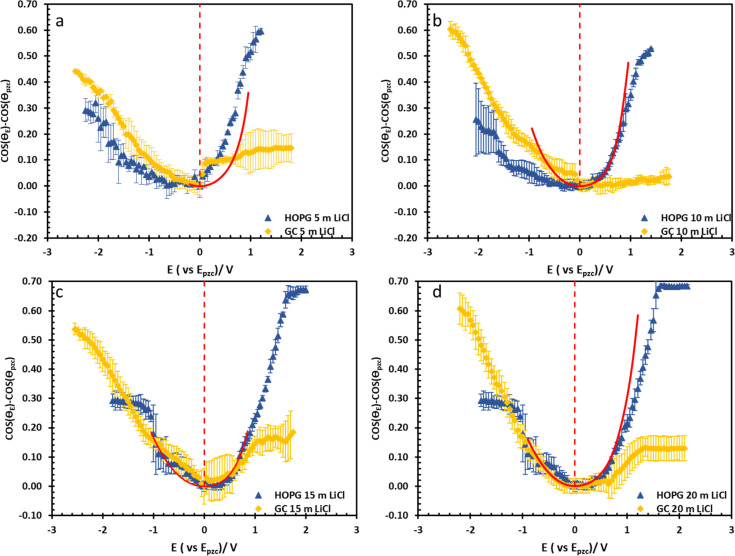
Electrowetting data from the Experimental section on HOPG (blue triangle) and GC (yellow rhombus) at LiCl concentrations of 5 m (a), 10 m (b), 15 m (c), and 20 m (d). The curves are plotted with the potential respect to *E*_pzc_ values which were estimated from electrowetting and EIS measurement, the red dashed lines refer to *E*_pzc_. The calculated data from Young–Lippmann equation base on calculated capacitance from EIS and measure surface tension of LiCl electrowetting on basal plane HOPG are shown in red solid line.

The substrate and electrolyte concentration effects on electrowetting are shown in Fig. 5, the experiment was performed for the droplet in air, using the set-up depicted in Fig. 1. The CA of the electrolyte droplet on carbon substrates was recorded as a function of potential (*E* with respect to Ag pseudo-reference electrode, see Fig. S2 in ESI†). The maximum change in CA (Fig. 4a) of the LiCl electrolyte on HOPG was found to be 60° (CA change from 73° at −0.7 V *vs.* Ag to 13° at 0.95 V *vs.* Ag) while the response on GC (Fig. 4b) was significant, but smaller than on HOPG, at 39° (CA change from 85° at −0.3 V *vs.* Ag to 46° at −2.5 V *vs.* Ag). Note that it was difficult to obtain consistent *E*_pzc_ between the two techniques: those from electrowetting were not exactly the same as the values from EIS analysis (Fig. 7). Thus, in order to match the experimental data with the theoretical Young–Lipmann relationship, the data are reported relative to the apparent *E*_pzc_ obtained from each technique. As LiCl concentration was decreased from 20 m to 5 m on HOPG, the electrowetting behaviour weakened and the response on the negative polarization deviated from the theoretical curve estimated from the Y–L equation (using capacitance measured from EIS analysis), the weak response and deviation in low concentration of LiCl in this experiment agrees with the EWOC response for KF electrolyte previously reported on HOPG.^11^ On the other hand, the plot at positive potential bias is still correlated with the capacitance data; this is because the faradaic process on positive polarization starts at a relatively high potential (>1.0 V *vs.* Ag), *i.e.* oxygen (OER) and chlorine (CER) evolution reactions, even at lower concentration (5 m LiCl for this experiment).^11^ Considering the shape of the asymmetric electrowetting curve on HOPG, the positive potential bias (*E* > *E*_pzc_) exhibits a stronger response than negative potential. Interestingly, the converse asymmetry was seen on the GC substrate. From this observation, we could assume that the Li^+^ cation was more strongly adsorbed on GC substrate while CI^−^ was preferentially adsorbed on HOPG: this is due to the fact that the decrease of CA caused by electrowetting comes from the decrease of liquid–solid interface tension caused by the accumulation of charge at the interface, *i.e.* EWOC is controlled by the interfacial capacitance and asymmetry in the latter should be reflected in asymmetry in the wetting plot.^5^

**Fig. 6:**
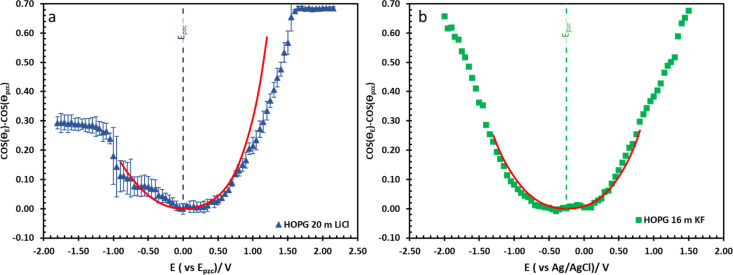
The comparison of electrowetting response of LiCl ((a), blue triangle) and KF ((b), green rectangle) at 20 m and 16 m on HOPG respectively, with the prediction line (red solid line) calculated from Young–Lippmann equation by using capacitance from EIS experiment and with measure surface tension from tensiometer. Data in (b) is reproduced from ref. 11.

The effect of electrolyte identity on electrowetting is shown in Fig. 6: the electrowetting response of 20 m LiCl is compared to 16 m KF^11^ on HOPG (Fig. 6), where the positive potential bias for both electrolytes exhibits similar electrowetting behaviour which is due to the fact that Cl^−^ is more chaotropic (more weakly hydrated) than F^−^,^41^ so it could be assumed that Cl^−^ ion dehydrates and penetrates to the interface easier. In contrast, the Li^+^ cation is more strongly hydrated than K^+^: for example, Tissandier *et al.* reported a lower solvation free energy of Li^+^,^42^ and Elliott *et al.* also performed an MD simulation showing that K^+^ could penetrate and be adsorbed on the inner Helmholtz layer at the graphene/electrolyte interface, while Li^+^ ions are adsorbed on the outer Helmholtz plane:^43^ a weaker capacitive response with 20 m LiCl on HOPG was consequently observed at negative potentials (Fig. 6a), which is consistent with earlier reports from our laboratory.^44^ Moving back to electrowetting on GC, the electrowetting curve also shows an asymmetric response, but the stronger response is seen on negative polarization (Fig. 5); the main factor behind this phenomenon is thought to be the different surface chemistry of GC, which possesses more edge plane exposure owing to its ribbon structure. This was also noted by McCreery and colleagues, who stated that the GC surface exhibited a higher density of edge planes on the surface and the edge density could be altered *via* various surface treatments, *i.e.* polishing, fracturing, and laser pulse.^45^ The GC surface thus contains a higher concentration of surface oxide species including hydroxyl, carboxyl, ester, and ketone which normally appear on the “zig-zag” edges. Moreover, the pH of the LiCl electrolyte evolves from slightly acidic at low concentrations, to 3.3 at the “water in salt” concentration (20.6 m).^46^ We hypothesise that there is a strong interaction of the Li^+^ ion with these oxygen-containing species when the negative potential bias was applied.

**Fig. 7:**
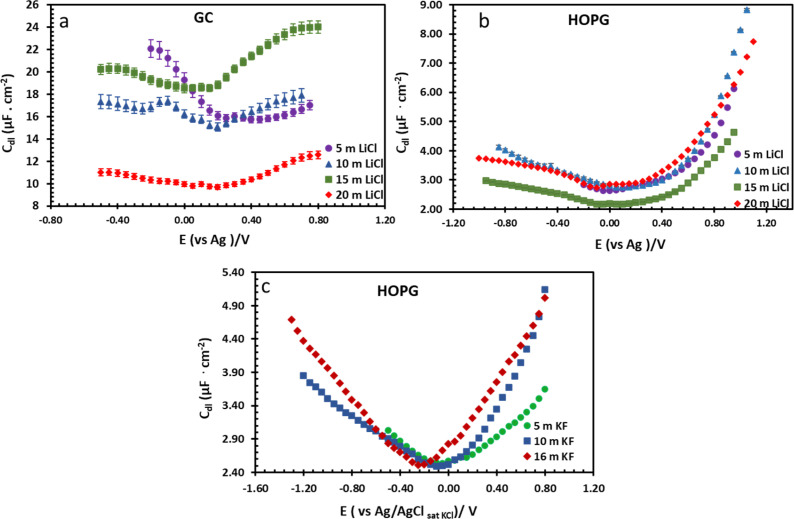
Potential dependence of capacitance of LiCl electrolytes in different concentrations on GC (a) and HOPG (b), and KF electrolyte on HOPG (c). The applied potential on all LiCl experiments were measured with respect to an Ag pseudo-RE, while the potentials in KF experiments were measured with respect to a Pt pseudo-RE. Capacitances of KF solutions were reproduced from ref. 11.

**Fig. 8:**
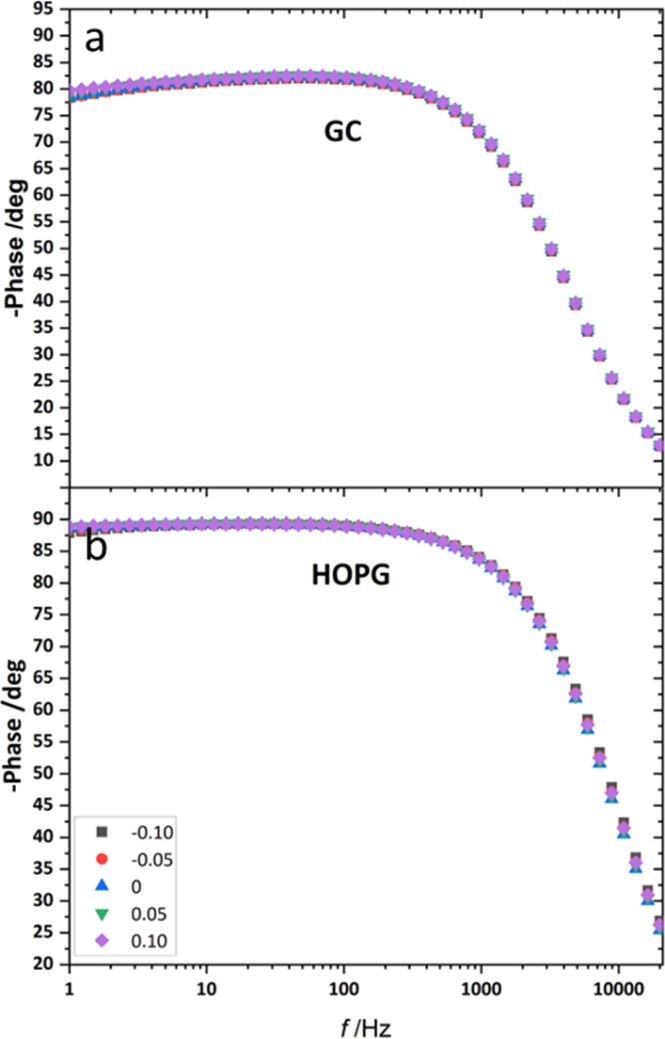
The example of Bode plot of 20 m LiCl on HOPG (a), and GC (b) from EIS analysis in the frequency range of 20k Hz to 1 Hz at applied potential from −0.10 V to 0.10 V (*vs.* Ag) with 0.05 V incremental step.

From the investigation of capacitance on HOPG and GC of LiCl solution within the whole concentration range used for the electrowetting experiments (Fig. 7a and b), the calculated capacitance values come from mean of the capacitance over the frequency range 10 Hz to 1k Hz, so as to ensure that the interface behaves close to an ideally polarizable electrode (phase angle close to −90°). The results show that the HOPG/LiCl interface exhibits an asymmetric parabola, unlike KF on HOPG which – as reported by Papaderakis *et al.* – yields a symmetric parabola.^44,47^ To return to the discussion on the likely origin of this phenomenon, as mentioned by Roget *et al.*, under the water-in-salt conditions, the larger ion (Cl^−^ in this case) forms a stronger bond with water (the solvent molecule) owing to the electrostatic interaction between the lone pair of water and the ion becoming stronger. For kosmotropic cations like Li^+^, the hydrogen bond between the cation and its water solvation shell was then strengthened in higher concentration. Thus, the asymmetric potential dependence of the capacitance indicates that the significant increase of capacitance at applied potentials positive of the potential of zero charge (*E* > *E*_pzc_) could be due to the weaker solvation shell around CI^−^ and water compared to Li^+^.^44^ The GC substrate also showed a similar trend in its capacitance curve at the higher concentrations (see more detail in Fig. S5†). Nevertheless, at the lowest concentration of LiCl (5 m in this experiment), GC exhibited the opposite trend: the specific adsorption of Li^+^ exhibited a significant increase for *E* < *E*_pzc_ owing to the absence of the ion pair effect. Moreover, *E*_pzc_ for each concentration was estimated from lowest value of the capacitance. The *E*_pzc_ of 5 m LiCl on GC and HOPG were determined to be 0.4 V and 0.0 V (*vs.* Ag) that corresponded to different specific adsorption of ions on these carbon materials found, as before in electrowetting in liquid/liquid configuration,^44^ and again indicative of a strong adsorption of Li^+^ on the GC surface. The *E*_pzc_ on GC then shifts negatively due to the increase of density profile of counter-ions at interface, when the electrolyte concentration rises. Interestingly, the opposite trend is seen for the HOPG substrate, regardless of the reported global minima (at −0.1 V *vs.* Ag) seen for the 20 m LiCl curve, which may be derived from Ag leakage from the quasi-reference electrode.^48^ Although the capacitances on the GC surface were relatively high, they agree with some earlier reports.^45,49^ The high magnitude of measured capacitance from the GC surface can be attributed to pseudo-faradaic processes, derived from the aforementioned oxide species as has been reported by Iamprasertkun *et al.* in their investigation of the edge plane capacitance of HOPG. The diffusional contribution to the pseudo-capacitance is reflected in the lower phase angle of the Bode plot on the GC substrate (approximately −82° while it almost reached −90° with basal plane HOPG) (Fig. 8a and b).

**Fig. 9:**
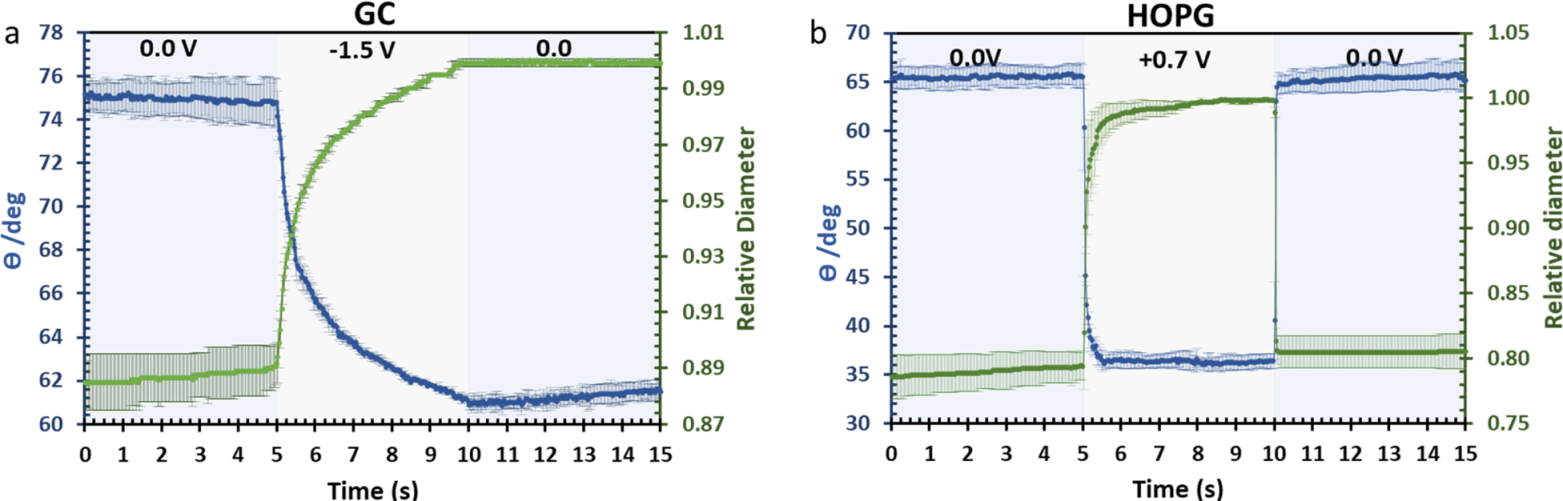
Dynamic electrowetting on GC (a) and HOPG (b) with negative and positive potential pulse *via* chronoamperometry technique respectively. The changes in CA (blue, left axis) and diameter size (green, right axis) of droplets are then recorded.

The investigation of dynamic wetting/de-wetting on the GC and HOPG substrates is summarized in the data of Fig. 9. From the advancing part, the CA on the GC substrate decreases only gradually when the potential bias was applied; the slightly rougher surface may make the formation of the new equilibrium three-phase contact line harder. It could be seen that after 0 V (*vs.* Ag) was reapplied to the electrolyte droplet, the droplet tried to rearrange itself (the CA increases to some degree). However, the relative droplet diameter remains constant; this suggests that the droplet remains on the surface in a Wenzel state,^34^ the pinning effect may then play a role on the irreversible change in CA,^50^ but it is surprising if the pinning effect alone is responsible for the irreversible behaviour seen, as the mean roughness of GC and HOPG are similar in magnitude (*vide supra*^23,24^). Another plausible factor is the higher density of edge planes on the GC surface due to its disorder (defective structure). In stark contrast, the HOPG surface, which is considered to be flat, still exhibits fully reversible electrowetting-dewetting on the timeframe of tens to hundreds of milliseconds. The time frame from this report is relatively high compared to the response from previous report by Papaderakis *et al.*^11^ The dynamic electrowetting in the previous report was conducted with 10 m KF solution while 20 m LiCl was used here, and the increase in concentration directly affects viscosity,^16,18^ so the charging and discharging process would take more time. Moreover, the applied potential bias from this study was 0.7 V *vs.* Ag (lower than applied potential was studied with KF at 1.5 V *vs.* Ag/AgCl), assuming that there was no electrooxidation and intercalation on the surface to affect advancing motion and delay receding motion. We note that the ratio between advancing and receding at 0 V to 0.7 V on HOPG with 20 m LiCl observed in this study, being a factor of 3, agrees with the previous report on 6 m LiCl stepping between potentials of −0.2 V (*vs.* Pt) and 0.7 V (*vs.* Pt).^29^

## Conclusions

In summary, this study has investigated electrowetting of highly concentrated LiCl electrolyte on two types of common carbon, namely HOPG and GC. Both substrates showed different specific adsorption on their surface; Li^+^ and Cl^−^ are strongly adsorbed on GC and HOPG respectively. These phenomena created an asymmetric electrowetting response on both substrates: this asymmetry is also apparent in the observed potential-dependent capacitance. From the study of dynamic electrowetting, GC exhibited an irreversible and gradual change in CA on electrowetting, while HOPG showed reversible and rapid changes.

## Author contributions

S. K.: conceptualization investigation, methodology, data curation, writing – review and editing. A. A. P.: investigation, data curation, and methodology. R. A. W. D.: conceptualization, methodology, resources, project administration, and writing – review and editing.

## Conflicts of interest

There are no conflicts to declare.

## Supplementary Material

NA-006-D4NA00506F-s001

## Data Availability

Original data will be made available on reasonable request.
