# Supplementary material for: Electrowetting on glassy carbon substrates
Source: Nanoscale Adv. 2024 Aug 30;6(21):5441–50. doi: 10.1039/d4na00506f (PMC11378016; doi:10.1039/d4na00506f)
Supplement: NA-006-D4NA00506F-s001 [file NA-006-D4NA00506F-s001.pdf]

Supplementary Information (SI) for Nanoscale Advances.
This journal is © The Royal Society of Chemistry 2024

Supporting information

Electrowetting on Glassy Carbon substrates

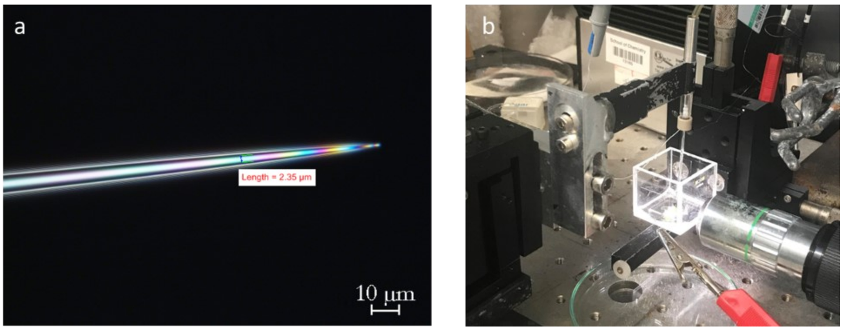

Figure S1: fabricated micropipette tip (a), and photograph of the setup used for electrowetting experiments (b).

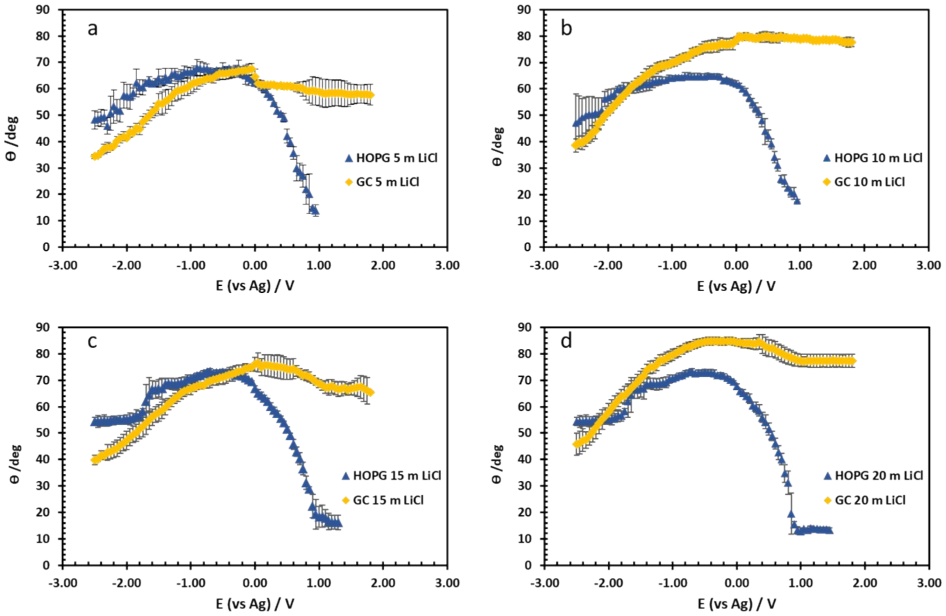

Figure S2: Potential dependence of contact angle for aqueous LiCl electrolyte, in air, using 5 m(a), 10 m(b), 15 m (c), and 20 m(d) on Glassy carbon (GC) and highly oriented pyrolytic graphite (HOPG) surfaces.

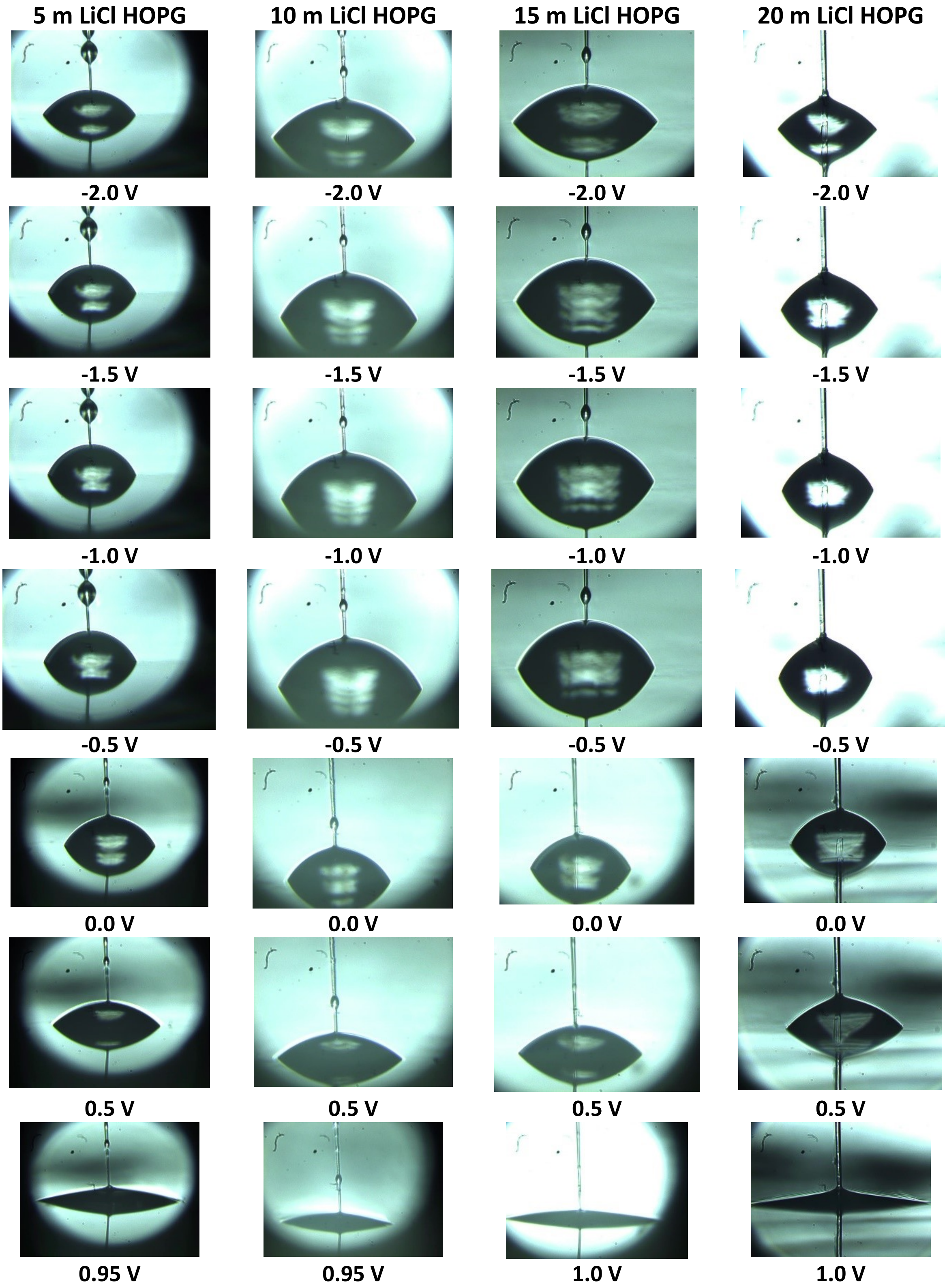

Figure S3. The droplet images with applied potentials respect to Ag taken during the electrowetting experiment on HOPG surface.

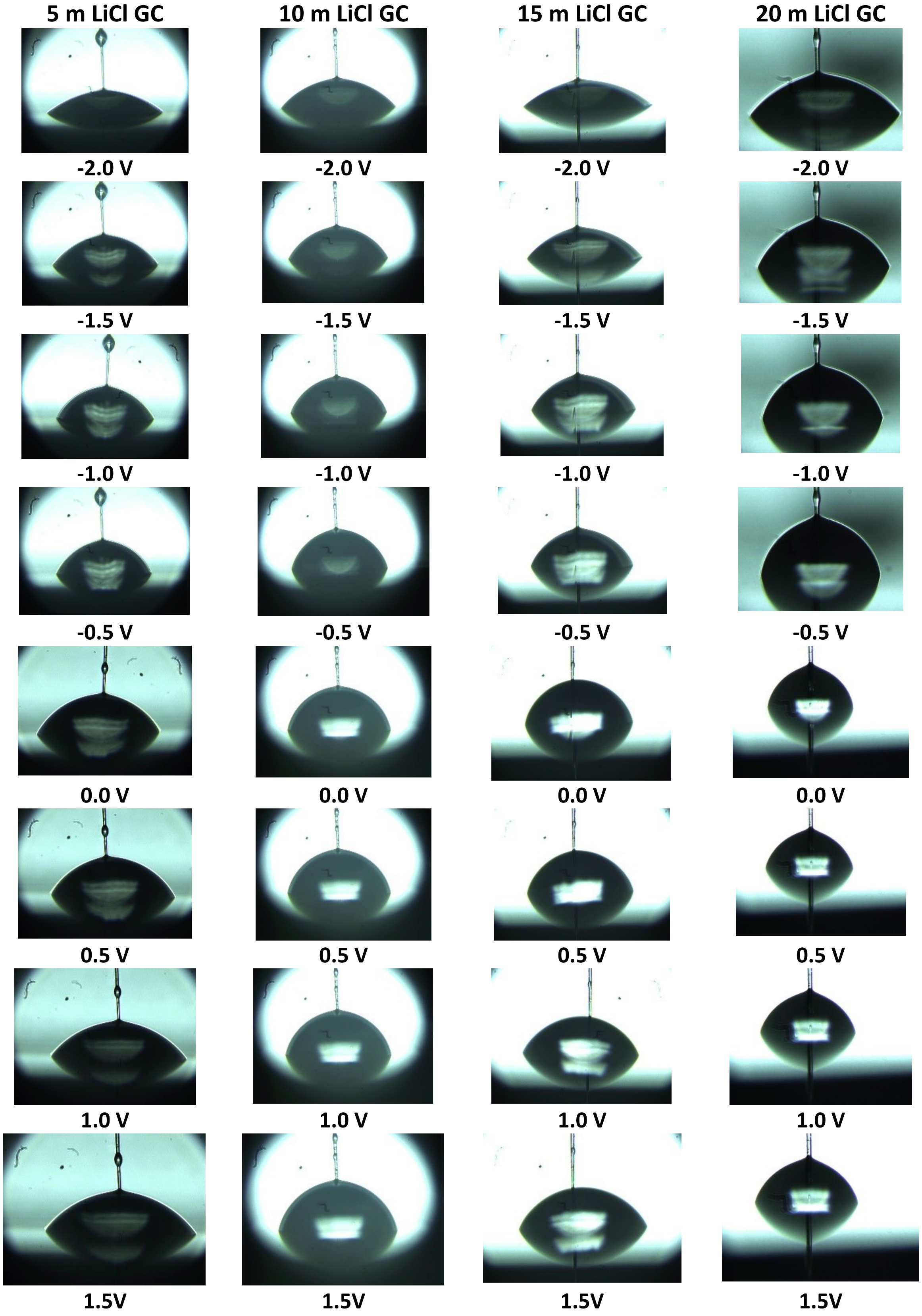

Figure S4.: The droplet images with applied potentials respect to Ag taken during the electrowetting experiment on GC surface.

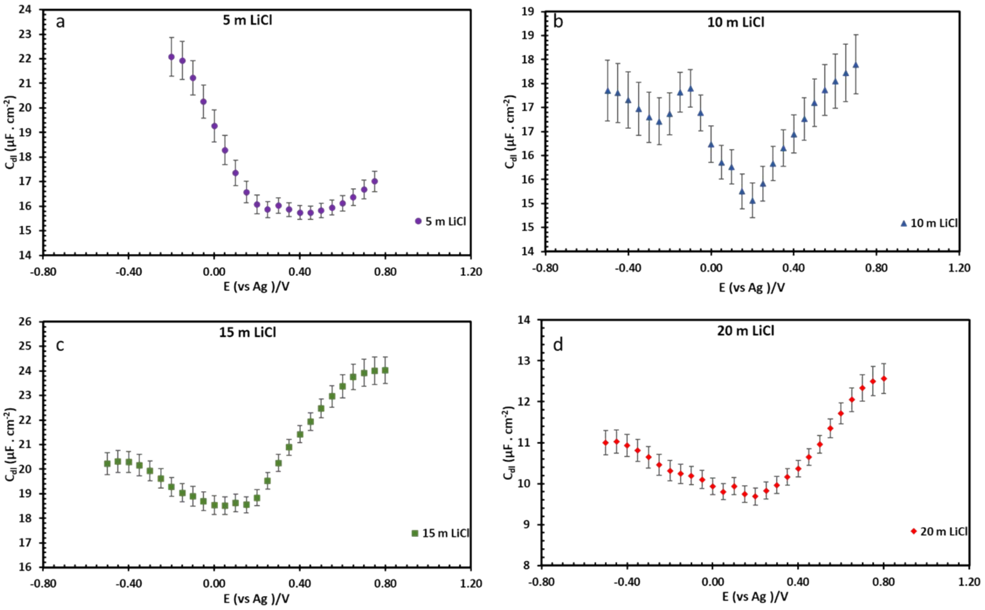

Figure S5: Calculated potential-dependent capacitance of 5 m (a), 10 m (b), 15 m (c), and 20 m (d) LiCl electrolyte on the GC surface.

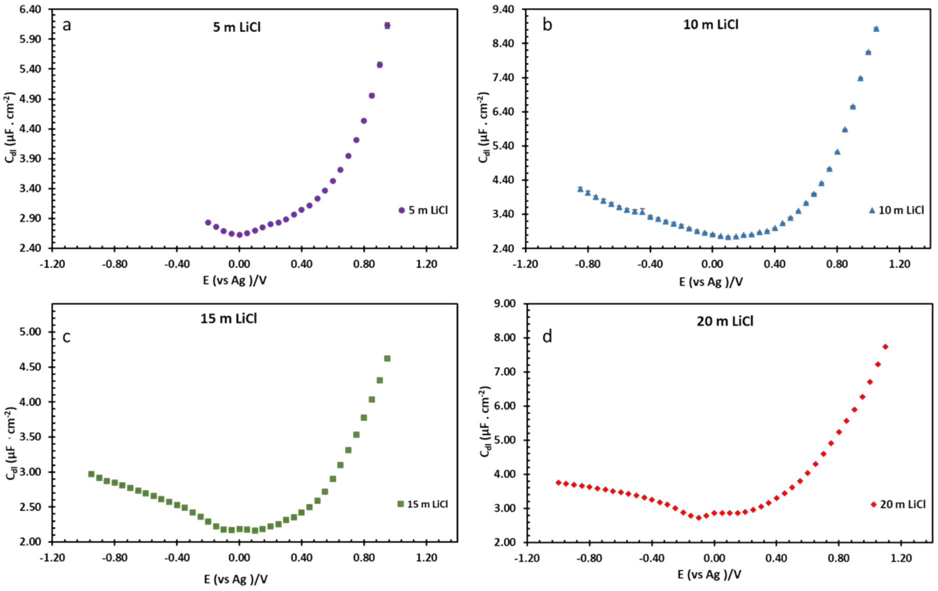

Figure S6: Calculated capacitance of 5 m (a), 10 m (b), 15 m (c), and 20 m (d) LiCl electrolyte on the HOPG surface.

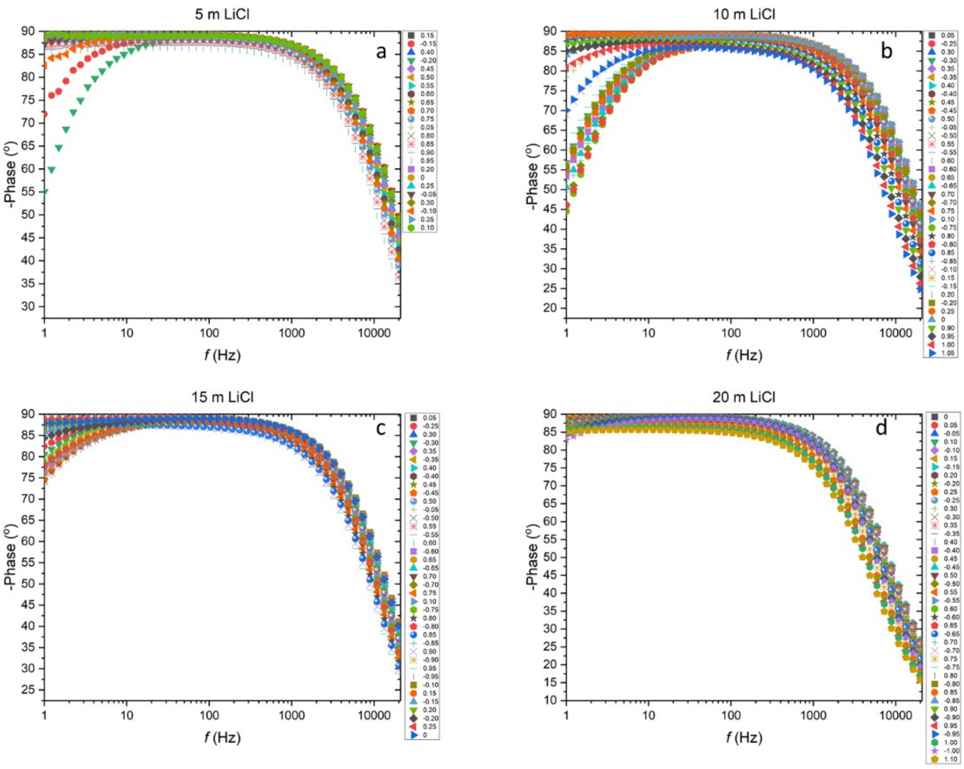

Figure S7: Bode plots of 5 m (a), 10 m (b), 15 m (c), and 20 m (d) LiCl solutions on HOPG with the frequency range of 20k-1 Hz, and 7 mV amplitude at potentials labelled in the legend of each plot.

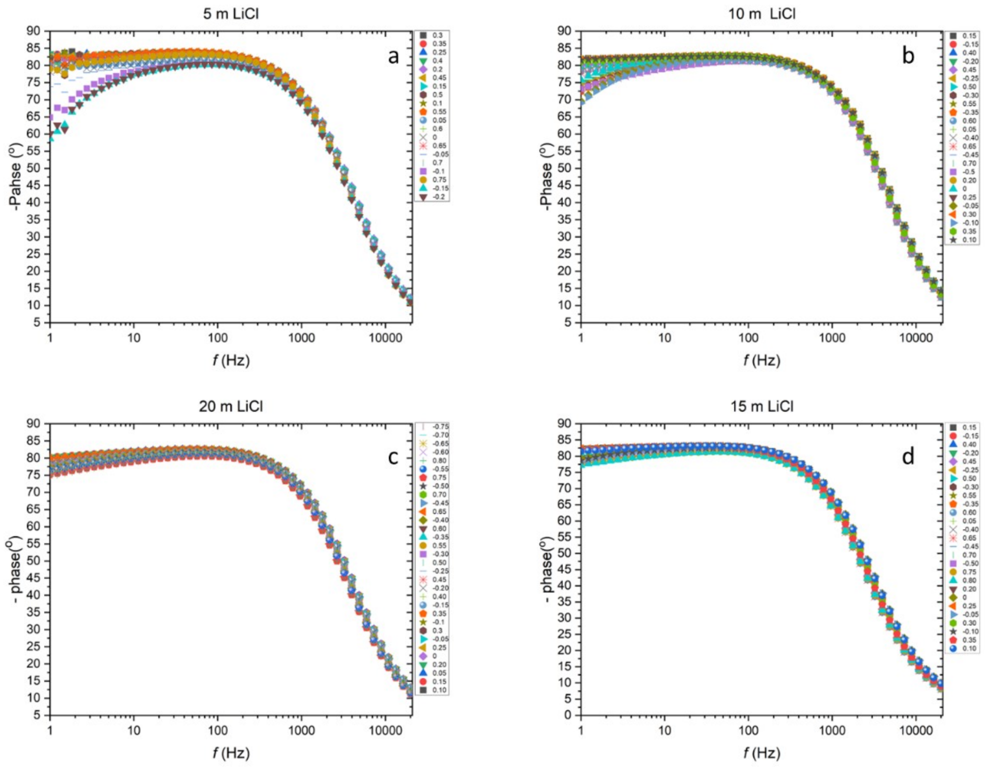

Figure S8: Bode plots of 5 m (a), 10 m (b), 15 m (c), and 20 m (d) LiCl solutions on GC surface with the frequency range of 20k-1 Hz, and 7 mV amplitude at the potentials given in the legend of each plot.
